# Acute sprint exercise transcriptome in adipose tissue of healthy adults: Associations with growth hormone

**DOI:** 10.14814/phy2.71160

**Published:** 2026-11-06

**Authors:** Mona Esbjörnsson, Håkan C. Rundqvist, Barbara Norman, Ted Österlund, Jens Bülow, Eva Jansson

**Affiliations:** ^1^ Division of Clinical Physiology, Department of Laboratory Medicine Karolinska Institutet Stockholm Sweden; ^2^ Unit of Clinical Physiology Karolinska University Hospital Stockholm Sweden; ^3^ Department of Clinical Physiology and Nuclear Medicine Bispebjerg University Hospital Copenhagen Denmark

**Keywords:** biopsy, high intensity, microarray analysis, sprint interval exercise, subcutaneous white adipose tissue, transcriptome

## Abstract

We hypothesised that acute sprint exercise induces changes in adipose tissue (AT) gene expression related to lipolysis, and that growth hormone (GH) contributes to this. Healthy males and females were randomised to perform 3 × 30‐s all‐out cycle sprints (SIT, *N* = 12) or unloaded cycling (CON, *N* = 6). AT‐biopsies were performed before and 2 h after exercise. Serum GH‐response was greater following SIT than CON, but no SIT‐specific enrichment of lipolysis‐related genes was found in a genome‐wide analysis. The hypothesis was partially supported. First, a group interaction was observed for the exercise‐induced change in GH‐receptor mRNA. Second, exercise‐induced changes in the pre‐selected lipolysis‐ and GH‐related genes *CISH, PTEN* and *G0S2* were associated with circulatory GH. In addition, in the genome‐wide analysis, changes in expression of *ARHGAP24, TAF4B, ARID5B, MIR604* and *MIR938* were associated with GH‐response. These genes have previously been found to be linked to AT‐metabolism. Among the most downregulated genes were core clock genes, which decreased independently of loading and likely explained by diurnal regulation. Sex influenced both the rate of GH‐response to exercise and the GH‐responsive gene *CISH*. This reinforces, from a molecular perspective, the importance of controlling for diurnal variation and sex in exercise studies on regulation of AT lipolysis.

## 1 INTRODUCTION

Sprint interval training (SIT) has been shown to reduce body fat mass or waist circumference (Poon et al., 2024; Viana et al., 2019), despite the low energy expenditure during a SIT session (Brownstein et al., 2022; MacInnis & Gibala, 2017). This may be explained by an increased rate of post‐exercise lipolysis and the thermogenic triglyceride‐free fatty acid (TG‐FFA) cycle, resulting in increased post‐exercise energy expenditure (Brownstein et al., 2022; Hazell et al., 2012; Panissa et al., 2021). Acute SIT is known to increase plasma catecholamines, growth hormone (GH) and interleukin‐6 (IL‐6) (Esbjörnsson et al., 2009; Esbjörnsson, Norman, et al., 2024), all potential candidates for increased lipolysis and TG‐FFA cycle related thermogenesis in adipose tissue (AT) during post‐exercise recovery (Enevoldsen et al., 2007; Lange, 2004; Laurens et al., 2020; Rognstad & Katz, 1966; Thompson et al., 2012; Yoshioka et al., 2001). Measurements of plasma glycerol from blood draining subcutaneous abdominal AT have confirmed that SIT stimulates lipolysis in adipose tissue (Esbjörnsson, Norman, et al., 2024).

Recently, we tested the hypothesis that post‐exercise expression of genes related to fat and energy metabolism would increase in response to SIT. However, this hypothesis was not confirmed by evaluating AT biopsies performed 15 min after the last sprint. On the contrary, the expression of such genes decreased (Esbjörnsson, Rundqvist, et al., 2024). Notably, GH has a delayed lipolytic effect, demonstrated both at rest after a bolus injection of GH (Høyer et al., 2020) and after exercise, with lipolysis starting around 1–2 h post‐exercise (Enevoldsen et al., 2007). This delayed action suggests that SIT‐induced GH release may drive sustained lipolysis and thermogenesis in AT during the later phases of post‐exercise recovery. Moreover, GH increases the phosphorylation of the transcription factor STAT5 in AT, in a GH‐receptor (GHR) dependent manner (Høyer et al., 2020) and subsequently also the expression of GH‐responsive genes (Hjelholt et al., 2019; Møller et al., 2003; Yoshioka et al., 2001). Consequently, increases in the expression of genes related to fat and energy metabolism post‐exercise may occur later than 15 min, which was the time point previously studied (Esbjörnsson, Rundqvist, et al., 2024).

To capture this potential delayed GH‐mediated response, AT biopsies were performed 2 h post‐exercise in the present study. We included both men and women in equal proportions, as sex‐related differences in GH release and GH‐responsive genes have been reported (Pritzlaff‐Roy et al., 2002; Veldhuis, Erickson, et al., 2011; Veldhuis, Roelfsema, et al., 2011; Vestergaard et al., 2014; Wideman et al., 2002). For example, circulatory GH shows a faster early response to exercise in women than in men (Eliakim, Cooper, & Nemet, 2014; Eliakim, Nemet, et al., 2014; Engström et al., 1998; Esbjörnsson et al., 2009; Rundqvist et al., 2019; Wideman et al., 2002). Furthermore, a control group of both sexes was included to adjust for other drivers of gene expression in AT besides exercise, such as diurnal (circadian) rhythm (Loboda et al., 2009).

It was hypothesised that acute SIT induces an increase in post‐exercise gene expression in AT related to lipolysis and energy expenditure. This occurs at the time of the GH‐induced increase in lipolysis, approximately 2 h post‐exercise. A secondary hypothesis was therefore that exercise‐induced changes in expression of GH‐responsive genes are related to the increase in serum GH during the acute SIT session. This hypothesis was tested using both pre‐selected GH targets and a genome‐wide approach. For the targeted analysis, we selected the established downstream targets CISH, PTEN and G0S2, which are involved in GH signalling. CISH is known to act as a negative regulator of GH‐induced lipolysis, thereby preventing excessive lipolysis (Hjelholt et al., 2019; Møller & Jørgensen, 2009). GH regulates lipolysis indirectly by activating PTEN (Sasaoka et al., 2006) and inhibiting G0S2 (Nielsen & Møller, 2014). A second rationale for selecting these targets was that GH has previously been shown to regulate their gene expression in adipose tissue (AT), based on studies involving bolus injection of GH into AT (Hjelholt et al., 2019; Høyer et al., 2020).

## 2 METHODS

### 2.1 Subjects

A total of 18 healthy voluntary subjects (9 females and 9 males) participated in the study. The mean ± SD for age, height and body weight are presented in Table 1.

**TABLE 1:** Subject characteristics in SIT (6 females and 6 males; all‐out sprints) and CON (3 females and 3 males; unloaded exercise).

Sex	Females		Males		Statistics
Load	SIT	CON	SIT	CON	Sex, *p*‐value
Age (years)	24 ± 1	24 ± 2	25 ± 5	24 ± 3	NS
Body weight (kg)	70 ± 7	79 ± 6	75 ± 11	74 ± 5	NS
Height (cm)	167 ± 3	174 ± 2	179 ± 5	180 ± 10	0.003
Fat (%)	26 ± 4	28 ± 4	14 ± 4	16 ± 4	<0.001
Fat mass (kg)	19 ± 4	22 ± 5	10 ± 3	12 ± 2	<0.001
FFM (kg)	44 ± 5	50 ± 1	61 ± 12	58 ± 9	0.016
AI	16.8 ± 3	17.8 ± 1	16.8 ± 2	13.8 ± 2	NS
PP I (W)	611 ± 104		719 ± 205		NS
PP II (W)	592 ± 96		673 ± 196		
PP III (W)	622 ± 76		676 ± 163		
MP I (W)	451 ± 56		590 ± 138		0.042
MP II (W)	457 ± 51		589 ± 144		
MP III (W)	463 ± 45		595 ± 118		
PP I (W/kg)	8.7 ± 0.7		9.6 ± 2.1		NS
PP II (W/kg)	8.4 ± 0.7		8.4 ± 2.0		
PP III (W/kg)	8.9 ± 0.7		8.9 ± 1.2		
MP I (W/kg)	6.5 ± 0.5		7.8 ± 1.2		0.022
MP II (W/kg)	6.6 ± 0.6		7.8 ± 1.2		
MP III (W/kg)	6.6 ± 0.7		7.9 ± 0.8		
PP I (W/FFM)	11.9 ± 1.4		11.1 ± 2.3		NS
PP II (W/FFM)	11.5 ± 1.2		10.3 ± 2.1		
PP III (W/FFM)	12.1 ± 0.8		10.4 ± 1.3		
MP I (W/FFM)	8.8 ± 0.7		9.1 ± 1.1		NS
MP II (W/FFM)	8.9 ± 0.7		9.0 ± 1.1		
MP III (W/FFM)	9.0 ± 0.5		9.2 ± 0.7		

*Note*: Values are means ± SD.

Abbreviations: AI, activity index; FFM, fat free mass; MP, mean power; NS, non significant; PP, peak power.

Subjects were recruited from voluntary students enrolled in various study programmes at Karolinska Institutet. Inclusion criteria were healthy, non‐smoking young adults of both sexes who engaged in regular physical activity, but not at an elite or competitive level. Individuals taking regular medication, except oral contraceptives and female subjects in the menstrual phase were excluded from the study. An activity index (Jansson & Hedberg, 1991) with values ranging from 5.5 to 20.5 was calculated to estimate physical activity during leisure time; the mean and range for our subjects were 16.5 (11–19.5). The percentage of body fat, fat‐free body mass and fat mass was estimated from skinfold measurements of the triceps, biceps and subscapular regions (Durnin & Womersley, 1974), and are presented in Table 1. Subjects were fully informed about the procedures and potential risks before providing written informed consent. The study was conducted in accordance with the ethical principles of the Declaration of Helsinki (World Medical, 2013) and approved by the Ethics Committee at Karolinska Institutet (Dnr 2015/574‐31, 2015/1054‐32, 2015/1646‐32, 2015/2340‐32).

### 2.2 Experimental protocol

After a 60‐s warm‐up at a cycle ergometer at a load of 25% of the individual braking load at 60 rpm, subjects were randomised to perform repeated bouts of sprint exercise (SIT group; 6 females and 6 males) or unloaded exercise (CON group; 3 females and 3 males). Subjects were asked to refrain from strenuous exercise for 24 h before the experiment. They reported to the laboratory between 08:00 am and 09:30 after an overnight fast, except for the instruction to consume one to two slices of white bread and a glass of water 1 h before arrival. An indwelling catheter was inserted into an antecubital vein. 15 mL of blood was sampled in the supine position before a 60‐s warm‐up, 9 min after each bout of exercise and up to 2 h after the last bout of exercise (Figure 1). Subcutaneous abdominal adipose tissue biopsies were performed under local anaesthesia without adrenaline, using a Hepafix needle (Braun Medical, Melsungen, Germany): before bout 1 and 2 h after the last bout of exercise, on different sides of the umbilicus.

**FIGURE 1:**
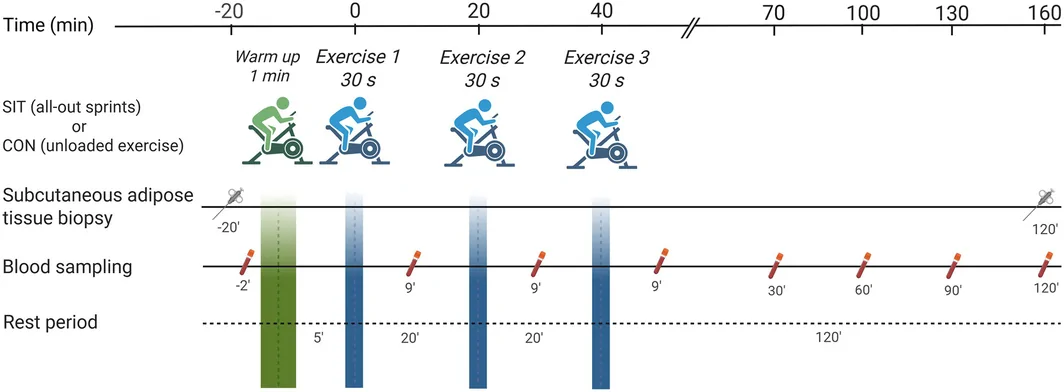
Schematic overview of the experimental protocol. Subjects were randomized after warming up to perform either 3 × 30‐s all‐out sprints (SIT group; 6 females and 6 males) or unloaded exercise (CON group; 3 females and 3 males). Created in BioRender. Rundqvist, H. (2026), https://BioRender.com/xkmfi67.

### 2.3 Adipose tissue blood flow

Subcutaneous abdominal adipose tissue blood flow was measured at rest and up to 2 h after the last sprint in 3 healthy, physically active subjects, using the same exercise protocol as in the SIT group in the current study (2 females and 1 male, age 20–25 years, BMI 19–28 kg × m^−2^), by the Xenon‐133 washout technique (Bülow & Madsen, 1976), the gold standard. However, it has unfortunately not been possible to access the isotope in Europe for several years. Therefore, we believe that this blood flow data should be reported, as further experiments with this method cannot currently be conducted. Although only 3 subjects were included, the results clearly show that blood flow decreases after the first sprint and thereafter increases to levels above rest, providing important information about possible stimuli for changes in gene expression. The study was approved by the Ethics Committee at Karolinska Institutet (Dnr 2015/574‐31, 2015/1054‐32, 2015/1646‐32).

### 2.4 Exercise protocol

Subjects in the SIT group performed 3 × 30‐s all‐out cycle sprints (Bar‐Or, 1987) on a mechanically braked cycle ergometer (Monark Ergomedic 894 E, Sweden), with 20 min of rest between sprints. They were instructed to pedal as fast as possible at a load of 0.075 kilopond × kg body mass^−1^. Peak power (the highest 5‐s power) and mean power (the average power during the 30‐s cycle) were calculated for each of the three 30‐s cycle sprints. A 20‐min recovery period was used to ensure near‐complete restoration of power output between sprint bouts, as previously applied in our sprint exercise studies (Esbjörnsson, Rundqvist, et al., 2024). Subjects in the CON group performed 3 × 30‐s bouts of unloaded exercise on the cycle ergometer with 20 min of rest between each bout and were instructed to pedal at 60 rpm.

### 2.5 Adipose tissue biopsy preparation and analysis

Subcutaneous adipose tissue samples obtained before and after exercise were immediately blotted, placed on a plastic filter (Sefar Nitex 06‐210/233, Bigman AB, Sweden), rinsed in sterile saline, cut into smaller pieces and frozen in liquid nitrogen. Samples were stored at −80°C until processed. Frozen tissue samples (30–100 mg) were homogenised with a Polytron homogeniser dispenser (Kinematica AG, Lucerne, Switzerland), and RNA was isolated with a Qiagen RNeasy Lipid Mini Kit (Cat. No. 74804, Thermo Fisher Scientific Inc., Waltham, MA, USA), treated with DNase (Qiagen RNase‐Free DNase Set, Cat. No. 79254, Thermo Fisher Scientific Inc., Waltham, MA, USA), and quantified using a Nanodrop spectrometer (ND‐1000: NanoDrop®, Thermo Fisher Scientific, Wilmington, DE, USA). Total RNA quality was assessed with an Agilent 2200 Tapestation (Agilent Technologies, Santa Clara, CA, USA).

Microarray analysis was performed using the Human Gene 2.1 ST Array (Cat. No. 902138, Thermo Fisher Scientific Inc., Waltham, MA, USA,). 150 ng of total RNA was used to generate amplified sense strand cDNA (sscDNA) targets with the GeneChip® WT Plus Reagent Kit (Cat. No. 902281, Thermo Fisher Scientific Inc.). 5.2 μg of fragmented and labelled sscDNA target was hybridised to the Human Gene 2.1 ST Array WT, followed by washing, on a GeneTitan MC instrument (Thermo Fisher Scientific), according to the manufacturer's protocol. Scanning was performed with the Affymetrix Gene Chip Scanner 3000 7G. Raw intensity data (CEL files) were processed using Affymetrix Transcriptome Analysis Console (TAC) Software, including the Signal Space Transformation—Robust Multi‐Chip Analysis (SST‐RMA) algorithm. The median RNA integrity (RIN) value was 7.7 (range 5.1–8.4). Ten of the 36 samples had RIN values between 6 and 7, and one had a value of 5.1, indicating slight RNA fragmentation in these samples. However, the Human Gene 2.1 ST array, used in this study, is a whole‐transcript array specifically designed to handle samples with some degree of RNA fragmentation. Furthermore, as part of post‐hybridisation quality control (QC), principal component analysis (PCA) was performed based on the 1000 genes with the highest variation across samples. No samples were identified as outliers in the PCA, and all samples were therefore retained for subsequent gene expression analyses.

Differentially expressed genes in response to exercise in the SIT and CON groups were identified using *limma*, which fits linear models to gene‐expression data, accounts for variability and adjusts for multiple testing (Ritchie et al., 2015). For the analysis of interaction in response to exercise between SIT and CON, robust regression from *limma* was used. QIAGEN's Ingenuity® Pathway Analysis (IPA®, QIAGEN, Redwood City, CA, USA) was used to identify upstream regulators from the list of differentially expressed genes. The Human Gene 2.1 ST array was selected as the reference. Regression analysis was performed between post‐exercise gene expression and GH peak, adjusted for measurements at rest. All raw data (CEL files) have been deposited in NCBI's Gene Expression Omnibus (Edgar et al., 2002) and are accessible via GEO Series accession number GSE335793.

### 2.6 Blood preparation and analyses

Blood samples were divided into two portions. One portion was transferred into sodium‐heparinised tubes and immediately centrifuged at 2000 *g* (4°C) for 10 min. One‐millilitre aliquots of this plasma were frozen in liquid nitrogen and stored at −80°C. The other portion was transferred to a serum tube, stored at room temperature for 20 min, and then treated in the same way as the heparinised blood. Plasma lactate was analysed using a Radiometer ABL 800 Flex blood gas analyser (Berman & Beving Lab, Triolab, Gothenburg, Sweden), and plasma glucose by the hexokinase method with a Roche/Hitachi cobas c‐system (Roche Diagnostics GmbH, Mannheim, Germany). Serum free fatty acids (FFA) were analysed by an enzymatic colorimetric assay (FUJIFILM Wako Chemicals Europe GmbH, NEFA‐HR (2), Nordic Biolabs, Täby, Sweden), serum insulin by an electrochemiluminescence immunoassay (Cobas e602/e801, Roche Diagnostics GmbH, Germany), and serum growth hormone (GH) by the IMMULITE 2000 Growth hormone (hGH) chemiluminescent enzyme immunoassay (Siemens Healthcare Diagnostics Products Ltd., United Kingdom). Growth hormone (GH) was measured in serum using the IMMULITE® 2000 Growth Hormone (hGH) chemiluminescent enzyme immunoassay (Cat. No. L2KGRH2, Siemens Healthcare Diagnostics Products Ltd., United Kingdom).

### 2.7 Statistics and calculations

Most variables were normally distributed, except for serum GH, which was log2‐transformed to reduce skewness. Gene expression data were also log2‐transformed for the global gene expression analysis. Values in the text are given as mean and SD unless otherwise stated. *p*‐values were considered statistically significant at *p* < 0.05. All exercise‐induced changes in blood data and selected gene expression data were analysed using 3‐factor ANOVAs: time, load (SIT or CON) and sex. Depending on the outcome of these ANOVAs, 1‐ or 2‐factor ANOVAs were performed as sub‐analyses (see Results and statistical details in figure legends). Student's *t*‐test for paired observations or groups or Wilcoxon signed‐rank test was used when appropriate. The relationship between gene expression data and metabolic or hormonal variables was analysed using single or multiple linear regression analysis. Adipose tissue blood flow data were analysed by the Friedman test for 1‐factor repeated measures (only three time points can be included at a time due to the low number of subjects). Statistical analyses were performed using Stat View (version 5.0; SAS Institute Inc., Cary, NC, USA).

The integrated serum GH concentration over time was calculated as the area under the curve (AUC) using the trapezoidal method (Stokes et al., 2003). A post hoc sensitivity power analysis was conducted for multiple regression analyses, testing the hypothesis of association between changes in exercise‐induced changes in gene expression and GH, using G*Power, version 3.1.9.7 (Faul et al., 2009). Given the sample size of 18 subjects, an alpha level of 0.05, a statistical power of 0.80, one tested predictor (load × GH peak—pre) and three predictors in total (GH peak—pre, load and load × GH peak—pre), the analysis indicated a theoretical minimum detectable interaction effect size of *f* ^*2*^ = 0.50. However, because G*Power assumes a balanced design, and our study features an unbalanced group distribution (SIT = 12 and CON = 6) the true minimum detectable effect size exceeds 0.50. This means that the interaction analysis has limited power to detect small‐to‐moderate effects and is primarily sensitive to large effects. In addition, observed effect sizes for the interaction were calculated for each of the eight regression analyses (Selya et al., 2012). A post hoc sensitivity analysis for the 3‐factor ANOVA using G*Power 3.1.9.7 was also conducted. The model included load (SIT vs. CON) and sex as between‐subject factors and time (pre‐ vs. post‐exercise) as a within‐subject factor. Given the final sample size of 18 participants (SIT = 6 males and 6 females; CON = 3 males and 3 females), *α* = 0.05, 80% power, an assumed correlation of 0.50 between repeated measurements, and a nonsphericity correction of *ε* = 1, the analysis indicated a minimum detectable effect size of Cohen's *f* = 0.448. Thus, under the specified assumptions, the study had 80% power to detect relatively large effects involving the repeated‐measures factor time, including interactions such as time × sex and time × load × sex. However, because of the small sample size and unequal distribution across the SIT and CON, this sensitivity estimate should be interpreted cautiously. Non‐significant findings should not be interpreted as evidence for the absence of small or moderate effects.

## 3 RESULTS

### 3.1 Power development

Peak power (PP) and mean power (MP) for the three bouts in females and males in the SIT group are shown in Table 1. PP and MP did not change significantly across the three sprints. PP and PP per kg body mass did not differ between sexes, but MP and MP per kg body mass were significantly higher in males than in females, regardless of the three sprints.

### 3.2 Metabolic measurements

Plasma lactate increased in the SIT group during the exercise session and then returned towards baseline (1‐factor ANOVA time, *p* < 0.001). No significant change over time was found in the CON group (Figure 2a). There were no interactions or main effects of sex (Figure 2a).

**FIGURE 2:**
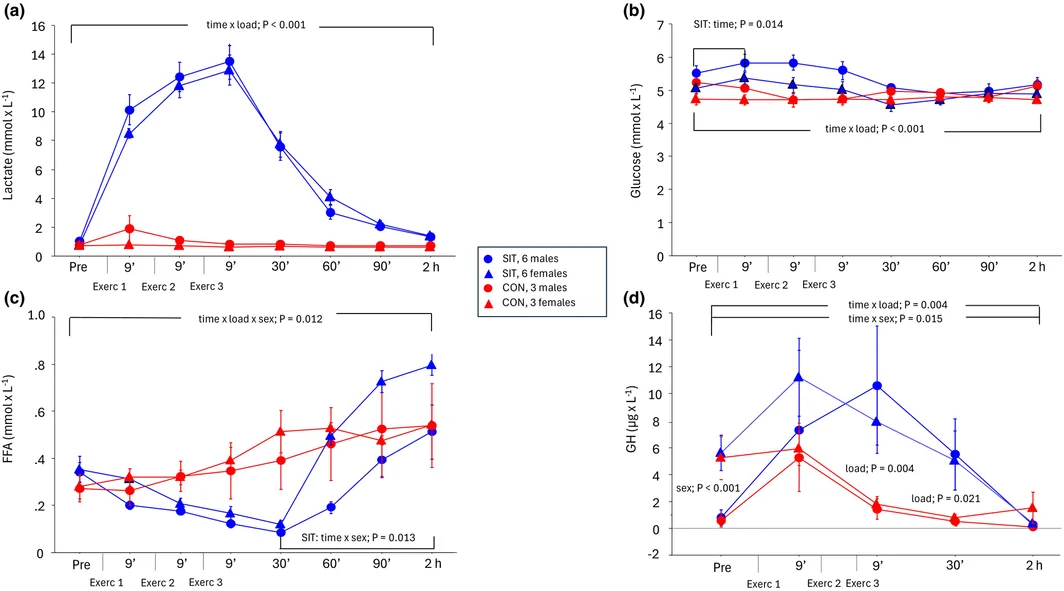
(a) Plasma lactate, (b) plasma glucose, (c) plasma FFA and (d) serum GH, pre ‐ exercise, during the exercise session (3 × 30‐s with 20 min rest in between) and 2 h after the last bout of exercise in SIT (sprint exercise; 6 males, 6 females) and CON (unloaded exercise; 3 males, 3 females). Data were analyzed by 3‐factor ANOVAs (time, load and sex) and by 1‐ or 2‐factor ANOVAs for further analysis serum GH data were log2‐transformed. Values are mean ± SE.

Plasma glucose increased in the SIT group by 6% between rest and 9 min after sprint 1 (*p* = 0.014), then gradually returned to baseline (1‐factor ANOVA time, *p* < 0.001). No significant change over time was found in CON (1‐factor ANOVA time, *p* = 0.150) (Figure 2b). There were no interactions or main effects of sex (Figure 2b).

Plasma FFA decreased in SIT by 60% during exercise (up to 30 min post sprint 3), independent of sex (2‐factor ANOVA time and sex, *p* < 0.001 for time). After the exercise session, FFA increased in SIT, with a greater increase in females than males (2‐factor ANOVA time and sex, *p* = 0.013 for time × sex, Figure 2c). In CON, plasma FFA increased over the exercise session, including the post‐exercise period, independent of sex (2‐factor ANOVA time and sex, *p* < 0.002 for time).

The integrated serum GH level over time, as estimated by AUC, was greater in SIT than in CON (*p* = 0.022), but did not differ between males and females. The GH peak ‐ pre (log2), used as the independent variable in the regression analysis presented below, was higher in SIT than in CON (*p* = 0.055 with *t*‐test and *p* = 0.001 with Wilcoxon signed‐rank test), but did not differ between the sexes. However, the females reached the GH peak earlier than males. In SIT, GH peak was reached after sprint 1 in females and after sprint 3 in males (2‐factor ANOVA: time and sex; *p* < 0.031 for time × sex) (note, GH was not analysed after the second bout of exercise). In CON, the GH peak was reached after the first exercise bout (unloaded exercise) in males, and in females, already at rest. A significant association was found between GH peak and FFA 2 h after the last sprint (*R* ^2^ = 0.48, *p* = 0.001) (Figure S1).

Serum insulin increased 3‐fold during the exercise session (measured only at rest and 2 h post sprint 3) in SIT (8 ± 2 vs. 24 ± 13 mIE × L^−1^; *p* = 0.001). No significant change was found in CON (7 ± 3 vs. 6 ± 2 mIE × L^−1^; *p* = 0.160). There were no interactions or main effects of sex in a 3‐factor ANOVA (time, load, and sex).

### 3.3 Subcutaneous adipose tissue blood flow

Subcutaneous adipose tissue blood flow decreased from rest to after sprint 1 in all 3 subjects (*p* = 0.050), then gradually increased until the end of the experiment, 2 h after the last sprint (Figure 3). Each data point for flow measurements (pre‐exercise, during the rest period between each of the three sprints, and at 2 h after the last sprint) represents the mean of repeated flow measurements over 15 min at steady state.

**FIGURE 3:**
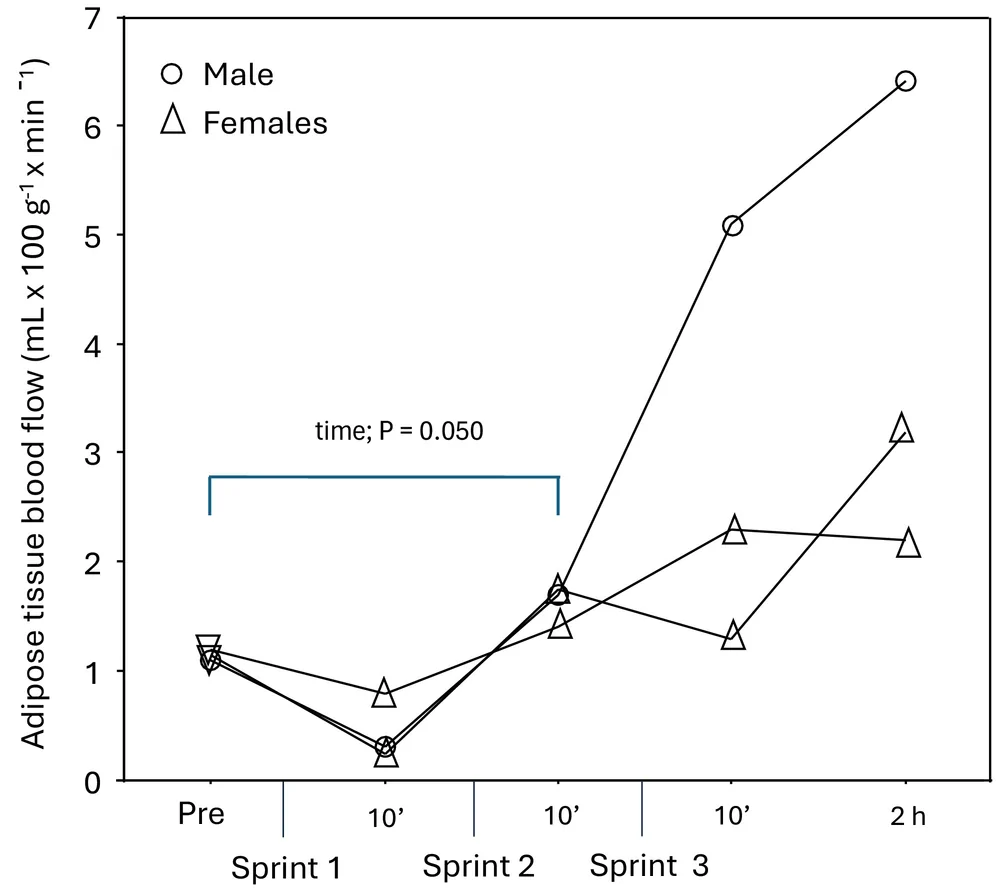
Abdominal subcutaneous adipose tissue blood flow pre‐exercise, during the exercise session (3 × 30‐s sprint with 20 min rest in between) and 2 h after the last bout of exercise in 3 subjects. Each data point in the figure represents the mean of repeated flow measurements over 15 min at steady state. The Friedman test is used for 1‐factor repeated measures.

### 3.4 Adipose tissue differential gene expression

To test our first hypothesis, that acute SIT induces an increase in post‐exercise adipose tissue gene expression related to enhanced lipolysis and energy expenditure, differentially expressed genes (DEGs) were analysed from the microarray. In the SIT group of 12 subjects, 34 differentially expressed genes were identified, with 13 upregulated (e.g. *MIR604, MIR938, ST6GALNAC3 and BMP4*) and 21 downregulated (e.g. *CTGF (CCN2), PER1, NEDD9* and *NFKBIA*), with an adjusted *p* < 0.1 (Figure 4, Table 2, Table S1).

**FIGURE 4:**
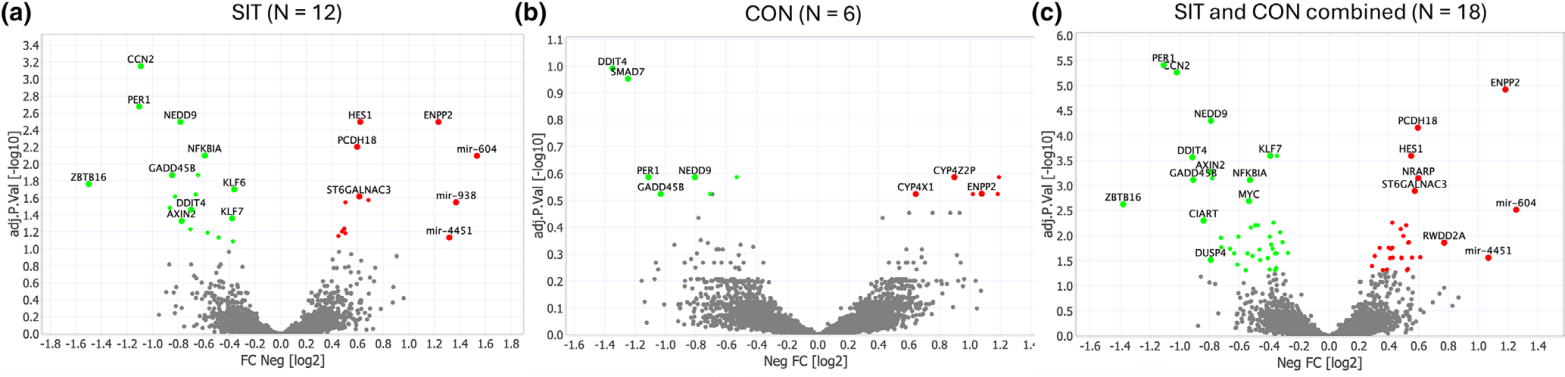
Volcano‐plots (a) SIT (12 subjects; cut off at adjusted *p* < 0.1), (b) CON (6 subjects; cut off at adjusted *p* < 0.3), (c) SIT and CON combined (18 subjects; cut off at adjusted *p* < 0.05) showing log2‐fold change (FC Neg) for post‐exercise/pre‐exercise (*x*‐axis) along with −log10 adjusted *p*‐value (y‐axis) from the differential gene expression analysis with *limma*. Downregulation is to the left in green and upregulation to the right in red.

**TABLE 2:** Differentially expressed genes expressed as fold change (FC; post/pre; FC <1 is expressed as neg FC) in subcutaneous adipose tissue in SIT (6 females and 6 males) and CON groups (3 females and 3 males). Data were analysed by *limma*.

SIT upregulated				SIT downregulated			
FC	Adj.*p*	Probeset	Gene symbol	FC	Adj.*p*	Probeset	Gene symbol
2.89	0.008	16712934	*MIR604*	−2.83	0.017	17117533	*ZBTB16*
2.58	0.028	16712936	*MIR938*	−2.15	0.002	16840846	*PER1*
2.49	0.074	16968521	*MIR4451*	−2.13	0.001	17023646	*CTGF*
2.35	0.003	17080554	*ENPP2*	−2.06	0.059	17007851	*LOC105375032*
1.61	0.027	17100420	*NRARP*	−1.84	0.012	16731441	*ZBTB16*
1.54	0.003	16949759	*HES1*	−1.82	0.033	16788618	*SNORD114‐2*
1.53	0.024	16666326	*ST6GALNAC3*	−1.80	0.014	16856803	*GADD45B*
1.51	0.006	16979875	*PCDH18*	−1.77	0.024	16958638	*KLF15*
1.42	0.028	17094814	*TMEM2*	−1.72	0.003	17015652	*NEDD9*
1.42	0.066	16709333	*TCF7L2*	−1.72	0.003	17118190	*NEDD9*
1.41	0.058	16848264	*ABCA10*	−1.71	0.047	16847933	*AXIN2*
1.39	0.062	16793129	*BMP4*	−1.63	0.059	16733516	*ADAMTS15*
1.37	0.071	16837366	*LINC01483*	−1.62	0.035	16705961	*DDIT4*
				−1.58	0.023	16834523	*AOC4P*
				−1.56	0.014	16681304	*ERRFI1*
				−1.51	0.008	16791991	*NFKBIA*
				−1.49	0.065	16871235	*CEBPA*
				−1.40	0.074	17072669	*MYC*
				−1.30	0.044	16907623	*KLF7*
				−1.30	0.082	16801789	*TPM1*
				−1.29	0.020	16711280	*KLF6*

CON upregulated				CON downregulated			
FC	Adj.*p*	Probeset	Gene Symbol	FC	Adj.*p*	Probeset	Gene Symbol
2.28	0.259	16753543	*MIR548C*	−2.55	0.102	16705961	*DDIT4*
2.27	0.299	16702827	*TMEM236*	−2.37	0.111	16855127	*SMAD7*
2.11	0.299	17080554	*ENPP2*	−2.16	0.259	16840846	*PER1*
2.03	0.299	17060442	*MIR25*	−2.04	0.299	16856803	*GADD45B*
1.86	0.259	16686734	*CYP4Z2P*	−1.75	0.259	17015652	*NEDD9*
1.56	0.299	16664388	*CYP4X1*	−1.75	0.259	17118190	*NEDD9*
				−1.63	0.299	16838169	*SEC14L1; SNHG20*
				−1.62	0.300	16935090	*FAM227A*
				−1.45	0.259	16861997	*ZFP36*

*Note*: Adjusted *p* < 0.1 for SIT and < 0.3 for CON.

Abbreviation: Adj.*p*, Adjusted *p*‐value.

In the CON group of 6 subjects, none of the genes met the cut‐off of adjusted *p* < 0.1, although fold changes for most of the DEGs in the SIT group exercise were of similar direction and magnitude as in the CON group (Figure S2, Table 2, Table S2). Interactions (time × load) were found for only 15 genes, of which 8 were uncharacterised (Table S3). Among the characterised genes, *GHR* was notable, as the secondary hypothesis was that the exercise‐induced response of specific GH‐responsive genes and the exercise‐induced increase in serum GH were associated.

Due to the few interactions observed, DEGs were also analysed independently of loading condition in all 18 subjects, where 74 differentially expressed genes were found, with 30 upregulated and 44 downregulated at an adjusted *p* < 0.05 (Table 3, Table S4). Some of the most downregulated genes were clock genes or genes regulated by clock genes, such as *PER1, NR1D1*, and *CIART*, and the clock‐regulated gene CTGF (Figure 5, Table 3), all of which decreased independent of load or sex; unadjusted *p* < 0.001 for time for all four genes. Adjusted *p*‐values for the downregulation of *PER1, NR1D1*, *CIART* and CTGF independent of group from *limma* were *p* < 0.001, *p* = 0.092, *p* = 0.005, and *p* < 0.001, respectively (Table 3, Table S4).

**TABLE 3:** Differentially expressed genes expressed as fold change (FC; post/pre, FC < 1 is expressed as neg FC) in subcutaneous adipose tissue in SIT and CON combined (9 females and 9 males). Data were analysed by *limma*.

Upregulated				Downregulated			
FC	Adj.*p*	Probeset	Gene symbol	FC	Adj.*p*	Probeset	Gene symbol
2.38	0.003	16712934	*MIR604*	−2.60	0.002	17117533	*ZBTB16*
2.27	0.000	17080554	*ENPP2*	−2.20	0.001	17007851	*LOC105375032*
2.10	0.027	16968521	*MIR4451*	−2.15	0.000	16840846	*PER1*
1.71	0.014	17010692	*RWDD2A*	−2.03	0.000	17023646	*CTGF*
1.53	0.027	16787135	*FLRT2*	−1.89	0.000	16705961	*DDIT4*
1.51	0.001	17100420	*NRARP*	−1.88	0.001	16856803	*GADD45B*
1.51	0.000	16979875	*PCDH18*	−1.79	0.005	16670479	*CIART*
1.49	0.001	16666326	*ST6GALNAC3*	−1.78	0.000	16731441	*ZBTB16*
1.47	0.027	17005276	*SOX4*	−1.73	0.001	16847933	*AXIN2*
1.46	0.000	16949759	*HES1*	−1.73	0.030	17075973	*DUSP4*
1.45	0.014	16664388	*CYP4X1*	−1.73	0.000	17015652	*NEDD9*
1.44	0.014	16683377	*ID3*	−1.73	0.000	17118190	*NEDD9*
1.44	0.046	16955935	*LOC100508226*	−1.72	0.001	16958638	*KLF15*
1.44	0.049	17087779	*NIPSNAP3B*	−1.65	0.011	17002846	*DUSP1*
1.43	0.006	17094814	*TMEM2*	−1.65	0.017	16855127	*SMAD7*
1.41	0.010	16776580	*ARHGEF7‐IT1*	−1.58	0.018	17002922	*LINC01484*
1.40	0.028	16971019	*GAB1*	−1.55	0.023	16733516	*ADAMTS15*
1.40	0.027	16947148	*ARHGEF26*	−1.53	0.038	17074815	*LONRF1*
1.39	0.007	17074567	*SOX7*	−1.52	0.010	17089502	*SLC25A25*
1.34	0.027	16942476	*MAGI1‐AS1*	−1.47	0.049	17094459	*AQP7P1*
1.34	0.017	16793129	*BMP4*	−1.46	0.023	16681304	*ERRFI1*
1.34	0.005	16709333	*TCF7L2*	−1.45	0.002	17072669	*MYC*
1.33	0.018	16961616	*TNFSF10*	−1.44	0.001	16791991	*NFKBIA*
1.33	0.028	17056137	*HOXA5*	−1.44	0.007	16834523	*AOC4P*
1.32	0.017	16899970	*LOC285074*	−1.43	0.026	16881838	*HK2*
1.31	0.047	16835604	*NXPH3*	−1.41	0.006	16952414	*CSRNP1*
1.28	0.049	17013033	*ECT2L*	−1.39	0.006	16861997	*ZFP36*
1.27	0.018	16662475	*AGO3*	−1.38	0.019	16871235	*CEBPA*
1.24	0.026	16751687	*ZNF740*	−1.38	0.031	16757800	*TMEM233*
1.22	0.040	16907198	*FAM126B*	−1.33	0.028	16952280	*SCN5A*
				−1.32	0.047	16811192	*TLE3*
				−1.32	0.010	17007701	*ANKS1A*
				−1.31	0.000	16907623	*KLF7*
				−1.31	0.015	16871072	*C19orf12*
				−1.30	0.018	16990949	*MIR143; MIR145; CARMN*
				−1.29	0.005	16801789	*TPM1*
				−1.28	0.023	16935427	*TOB2*
				−1.28	0.049	16859851	*KLHL26*
				−1.28	0.044	17113147	*TSC22D3*
				−1.28	0.023	16887194	*NOSTRIN*
				−1.27	0.000	16711280	*KLF6*
				−1.25	0.009	16985094	*ZSWIM6*
				−1.24	0.014	16763540	*SLC38A2*
				−1.21	0.022	16778433	*ELF1*

*Note*: Adjusted *p* < 0.05.

Abbreviation: Adj.*p*, Adjusted *p*‐value.

**FIGURE 5:**
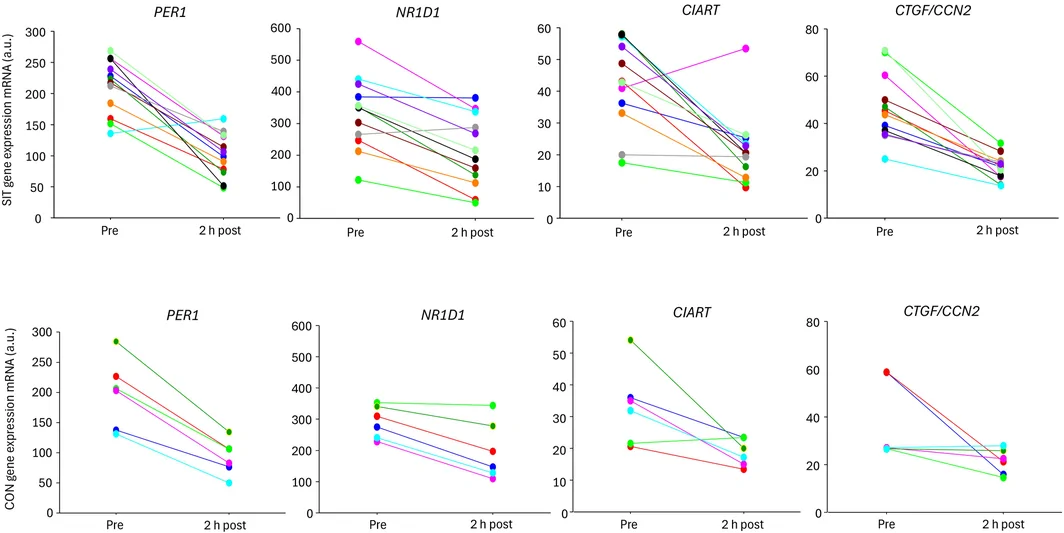
Individual values of core clock gene expression *PER1*, *NR1D1* and *CIART* and of *CTGF*, a downstream target of core clock genes, pre‐exercise and 2 h after the last bout of exercise in SIT (12 subjects) and CON (6 subjects).

### 3.5 Predicted anti‐inflammatory response

Predicted change (2 h post‐exercise ‐ pre) in activity state was identified by IPA Upstream Analysis. In the SIT group, a list of 77 characterised genes with adjusted *p*‐value <0.3 was analysed by IPA with a *z*‐value cut‐off at ±2 and *p*‐value cut‐off at *p* < 0.05 (Table S5). Only a few factors were predicted to be activated; for example, the transcription regulators SOX2, PAX5, and TCF12 and the kinase TGFBR2. However, several factors were predicted to be inactivated, such as the transcription regulators CREB1, LDB1, KLF6, SMAD3, NFKB1, and RELA, as well as cytokines including CSF1, CSF2, and TNF. Further examples of inactivated factors are the growth factors EGF and PDGF‐BB, the kinases PI3K and p38MAPK, the clock protein BMAL1 (*z* = 1.95), and the hormones aldosterone, progesterone, and insulin.

In the CON group, a list of 136 characterised genes with ±1.6 FC was also analysed by IPA with a *z*‐value cut‐off at ±2 and *p*‐value cut‐off at *p* = 0.05 (Table S6). As in the SIT group, only a few factors were upregulated. Among the inactivated factors, several were found to be regulated in both SIT and CON, such as SMAD3, NFKB1, CSF1, EGF, p38MAPK, aldosterone and insulin. This finding is consistent with only a few interactions in DEGs between SIT and CON. The pattern for the predicted changes of various factors in AT is consistent with decreased inflammation (e.g. NFKB1 and cytokines) and decreased adipogenesis (TGFBR2). Upstream Analysis (*z*‐value cut‐off at ±2 and *p*‐value cut‐off at *p* = 0.05) was also performed on the pooled data from a list of 110 characterised genes, *p* < 0.1 (Table S7) The outcome was similar to the separate group analysis, with few activated factors (e.g. TGFBR2 *z* = 2.4, *p* = 7 × 10^−3^ and GH *z* = 1.8, *p* = 2 × 10^−10^) and several inactivated factors indicating decreased inflammation, such as NFKB1, TGFB1 and various cytokines, growth factors and hormones.

### 3.6 Associations between adipose tissue gene expression and growth hormone

To test our secondary hypothesis regarding associations between exercise‐induced gene expression and GH, we performed regression analyses. First, we conducted a global regression analysis between post‐exercise gene expression (adjusted for pre‐exercise values) and serum GH peak (adjusted for pre‐exercise values). Both SIT (all‐out bouts) and CON (unloaded exercise) were included (*N* = 18). The genes with the lowest adjusted *p*‐values were *ARHGAP24* (*p* = 0.073), *TAF4B* (*p* = 0.114), *ARID5B* (*p* = 0.114), *MIR604* (*p* = 0.114) and *MIR938* (*p* = 0.114), all of which were directly related to the GH peak (Table S8). Given the exploratory nature of the global regression analysis and the relatively small sample size of this human study, we used an adjusted *p* ≤ 0.1 threshold (10% of the listed genes might be false positives) to identify candidate genes for further investigation. In contrast, the targeted analysis focused on established downstream targets of GH signalling (CISH, PTEN and G0S2; pre‐validated targets). For this hypothesis‐driven analysis, we used unadjusted *p*‐values to explore associations between the differential gene expression and GH response to exercise by the multiple regression analysis including load, GH peak ‐ pre and load × GH peak ‐ pre (Figure 6). To further describe and visualise the relationship between differential gene expression and GH response to exercise, the five top‐ranked genes in the global analysis were also included in the multiple regression analysis. The exercise‐induced changes in *ARHGAP24, ARID5B, TAF4B, MIR604, MIR938, CISH* and *PTEN* mRNA were found to be directly and *G0S2* mRNA inversely related to the exercise‐induced increase in GH (GH peak ‐ pre) (Figure 6). Interaction terms (load × GH peak ‐ pre) were included in the analyses above (Figure 6) to test whether the relationship between exercise‐induced gene expression and GH differed between SIT and CON. Given the small, unbalanced sample size (SIT = 12, CON = 6), the study had limited power to detect interaction effects (see Methods). Significant interactions were, however, observed for *MIR938* (*p* = 0.016, *f* ^2^ = 0.053), *CISH* (*p* = 0.036, *f* ^2^ = 0.38), and *PTEN* (*p* = 0.030, *f* ^2^ = 0.42), while *ARID5B* approached significance (*p* = 0.052, *f* ^2^ = 0.32) (Figure 6). For these genes, workload moderated the relationship between GH and gene expression, with greater gene expression responses for a given GH increase in SIT than in CON. In contrast, *MIR604*, *TAF4B*, *ARHGAP24* and *G0S2* showed no significant interaction, suggesting similar regression slopes between groups, with individuals in the CON group located in the lower half of the range of the X‐variable GH peak ‐ pre (Figure 6). Overall, the findings indicate a greater response in SIT for the GH‐related genes. However, the small and unbalanced sample size limited power to detect small‐to‐moderate interaction effects. Thus, the distinction between significant and non‐significant interactions should be considered exploratory, as subtle effects may have remained undetected. As a sensitivity analysis of the associations between exercise‐induced gene expression and GH, we also performed single regression analyses in the SIT group alone. The corresponding *R* ^*2*^ and *p*‐values are presented in the legend of Figure 6 and were mainly consistent with the findings from the combined analysis of all 18 participants in the multiple regression analyses. Using the integrated serum GH level over time, as estimated by AUC as the independent variable in the regressions presented in Figure 6 did not essentially change the outcome, because there was a strong association between GH peak—pre and AUC adjusted for baseline (*R* ^2^ = 0.855 for unlogged variables and *R* ^2^ = 0.829 for log2).

**FIGURE 6:**
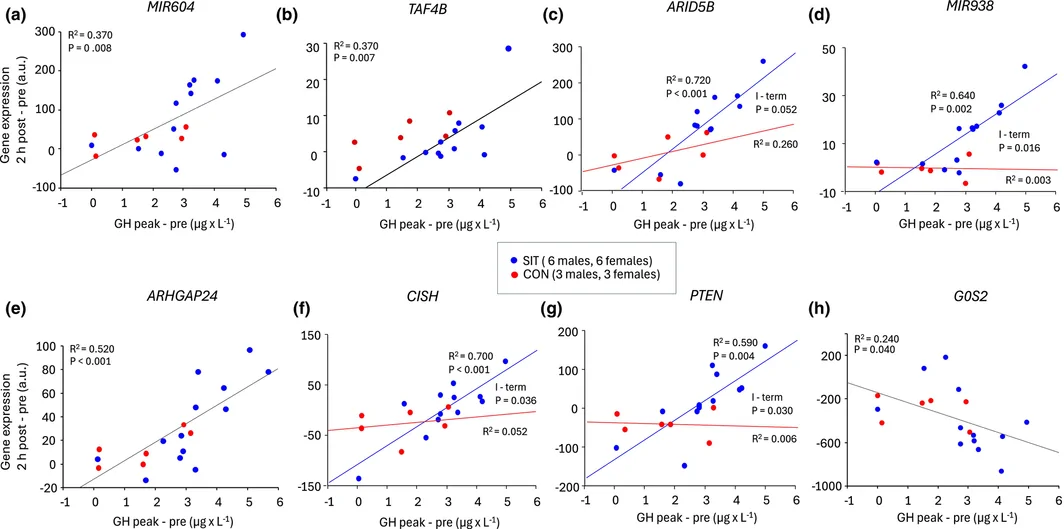
Exercise‐induced changes of gene expression (2 h post ‐ pre) related to increase in serum GH (GH peak ‐ pre) in SIT (12 subjects blue dots) and CON (6 subjects red dots) groups combined. The genes presented were those identified from the genome‐wide regression analysis (a–e) and the pre‐selected GH‐targets (f–h). Serum GH (GH peak ‐ pre) data were log2‐transformed. *R* ^2^‐values for SIT (12 subjects) only were *MIR604* (*R* ^2^ = 0.365, *p* < 0.037), *TAF4B* (*R* ^*2*^ *= 0.555, p* < 0.006), *ARID5B* (*R* ^2^ = 0.719, *p* < 0.001), *MIR938* (*R* ^2^ = 0.641, *p* < 0.002), *ARHGAP24* (*R* ^2^ = 0.538, *p* < 0.007), *CISH* (*R* ^2^ = 0.701, *p* < 0.001), and *PTEN* (*R* ^2^ = 0.587, *p* < 0.004) and *G0S2* (*R* ^2^ = 0.274, *p* < 0.080).

To analyse whether load or sex contributed to interindividual variation in the eight GH‐associated genes described above, 3‐factor ANOVAs (time, load, sex) were performed. time × load interactions (significant or approaching significance) were found for *TAF4B* (*p* = 0.060), *ARID5B* (*p* = 0.160), *MIR938* (*p* = 0.006), *CISH* (*p* = 0.046) and *PTEN* (*p* = 0.084), as well as time × load × sex for *MIR604* (*p* = 0.114) (Figure 7). Based on the outcome of the 3‐factor ANOVAs, 2‐factor ANOVAs were analysed separately for the SIT group. Time × sex interactions (significant or approaching significance) were found for *TAF4B* (*p* = 0.111), *MIR938* (*p* = 0.038), *MIR604* (*p* = 0.030), *CISH* (*p* = 0.003) and *PTEN* (*p* = 0.140), with males showing increased expression for *MIR938* (*p* = 0.003), *MIR604* (*p* = 0.008), *CISH* (*p* = 0.004) and *PTEN* (*p* = 0.094), but not females. All *p*‐values were unadjusted. These interactions indicate that both loading conditions and sex contributed to the interindividual variations in these GH‐associated genes. The sensitivity power analysis indicated that the study was primarily sensitive to relatively large effects. Accordingly, because of the small sample size and unequal distribution across the SIT and CON, this sensitivity estimate should be interpreted cautiously. Non‐significant findings should not be interpreted as evidence for the absence of small or moderate effects.

**FIGURE 7:**
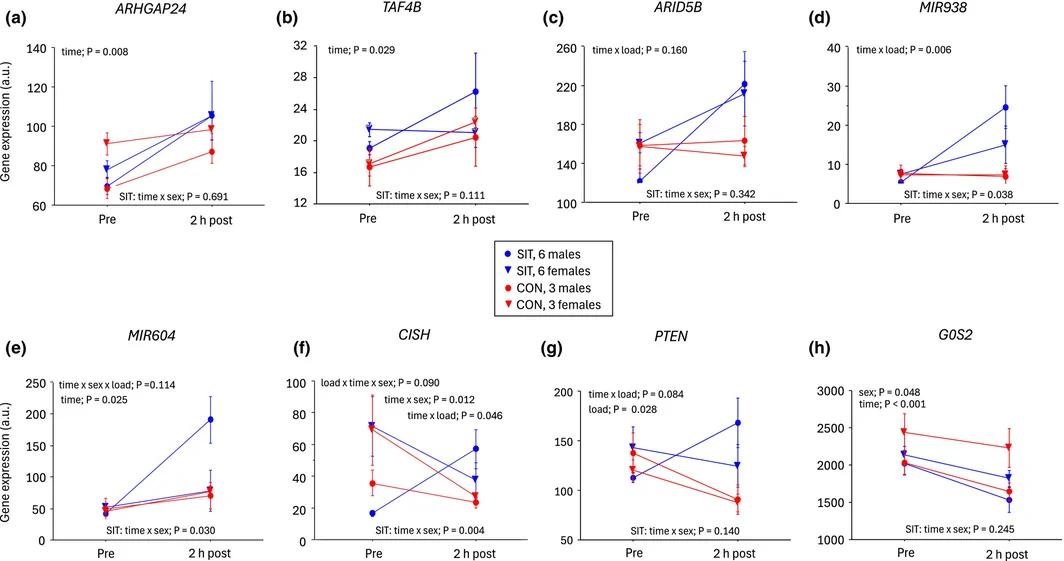
Gene expression pre‐exercise and 2 h post‐exercise divided by load (SIT, 6 females and 6 males) and (CON, 3 females and 3 males) and sex. The genes presented were identified from the genome‐wide regression analysis (a–e) and from the pre‐selected GH‐targets (f–h). Serum GH (GH peak ‐ pre) data were log2‐transformed. Values are mean ± SE.

## 4 DISCUSSION

### 4.1 Key findings

Circulatory growth hormone (GH), which increases lipolysis in abdominal subcutaneous adipose tissue (AT) (Kopchick et al., 2020) demonstrated a greater response to acute sprint interval exercise (SIT) than to unloaded bouts of exercise (CON). Despite this, only a few interactions were found in the differentially expressed genes (DEGs) in AT between SIT and CON in the genome‐wide approach. However, one of these genes was the growth hormone receptor (*GHR*), indicating a GH‐effect on AT that differed between SIT and CON. Nevertheless, we found no major post‐exercise enrichment of DEGs related to lipolysis or thermogenesis in AT. Yet, the exercise‐induced changes in gene‐expression of the pre‐selected GH‐targets *CISH*, *PTEN* and *G0S2* were associated with the exercise‐induced increase in serum GH. This is consistent with a GH‐mediated effect of SIT on lipolysis and thermogenesis, although it is important to note that lipolysis was not directly measured in the current study. In the genome‐wide approach, the exercise‐induced changes in gene‐expression of *ARHGAP24*, *TAF4B*, *ARID5B*, *MIR604* and *MIR938* were associated with the increase in serum GH. The function of these genes is not well understood, but some relationship to AT metabolism has been demonstrated for ARID5B (Whitson Jr. et al., 2021) and MIR604 (Lee, 2018; Weldy et al., 2020). Moreover, the upstream analysis of the DEGs suggested an anti‐inflammatory response to acute SIT. Surprisingly, a similar trend was also found in CON. Among the most downregulated genes, were the core clock genes *PER1*, *NR1D1* and *CIART*, which decreased similarly in both SIT and CON. Finally, some sex‐related differences were identified, especially at rest, for serum GH and the GH‐responsive gene *CISH* which is consistent with previous studies (Hjelholt et al., 2019; Høyer et al., 2020).

### 4.2 Core clock genes were altered independently of SIT or CON

An important and unexpected finding in this study was that similar fold changes in gene expression over time were observed in both SIT and CON. In particular, we wish to highlight the 2‐fold decrease in the core clock gene *PER1* mRNA, which was independent of group. A decrease was also observed for the other two clock genes, *NR1D1* and *CIART* mRNA, independent of group. *PER1* mRNA peaks at 8.30 am and its expression decreases throughout the day (Dreher et al., 2023; Loboda et al., 2009). Thus, our experiments were conducted during a period when *PER1* mRNA levels decline, which may explain our findings of decreased *PER1* gene expression, independent of SIT or CON. The similar changes in gene expression in SIT and CON in the present study raise important methodological questions. It is possible that the time of day is a stronger stimulus for changes in AT gene expression than SIT itself for certain genes. Indeed, it has been reported that diurnal rhythm is a significant driver of gene expression variations in human AT, with 25% of genes changing their expression over the day due to the diurnal rhythm (Loboda et al., 2009). Clock genes in AT regulate gene expression in energy metabolism. This reinforces, from a molecular perspective, the importance of controlling for diurnal variation in future exercise studies of AT, especially those related to lipolysis and thermogenesis. In fact, in a recent study it was reported that the expression in core clock genes such as *PER1*, *NR1D1* and *CIART*, decreased independent of exercise intensity (Ahn et al., 2025). The authors suggested that the non‐exercise factors such as fasting or diurnal variations could have influenced upon the exercise‐induced response as verified very recently in a BioRxiv preprint (Ahn et al., 2026).

### 4.3 Role of growth hormone in lipolysis and regulation of gene expression

The exercise‐induced increase in GH has previously been shown to have a delayed stimulatory effect on post‐exercise lipolysis (Enevoldsen et al., 2007) and, consequently, most likely also on post‐exercise free fatty acids (FFA) turnover, that is, thermogenesis (Rognstad & Katz, 1966; Townsend et al., 2017; Yoshioka et al., 2001). Our secondary hypothesis was, therefore, that exercise‐induced changes in the expression of GH‐responsive genes at 2 h post‐exercise were related to the increase in serum GH during the exercise session. The rationale for this was that GH activates the transcription factor STAT5 in AT and subsequently the expression of typical GH‐responsive genes (Hjelholt et al., 2019; Møller et al., 2003; Yoshioka et al., 2001) in a GHR‐dependent manner (Høyer et al., 2020). In the targeted analysis, we selected the genes *CISH*, *PTEN* and *G0S2* based on their roles in GH‐regulated AT lipolysis. CISH is known to be a negative regulator of GH‐induced lipolysis, suppressing STAT5 activation to avoid excessive lipolysis (Hjelholt et al., 2019; Møller & Jørgensen, 2009). GH also regulates lipolysis indirectly by activating PTEN, which in its turn suppresses antilipolytic insulin/Akt signalling (Sasaoka et al., 2006), and by inhibiting G0S2, which inhibits the enzyme adipose tissue lipase (Nielsen & Møller, 2014). A second reason for choosing these genes was that GH regulates their gene expression in AT, as based on earlier findings from bolus injection of GH into AT (Hjelholt et al., 2019; Høyer et al., 2020). We found that the exercise‐induced increases in *PTEN* and *CISH* mRNA were directly related to the increase in serum GH, whereas the decrease in *G0S2* mRNA was inversely related to the increase in GH (Figure 6). This is consistent with a GH‐mediated effect of SIT on lipolysis and thermogenesis, although it is important to note that lipolysis was not directly measured in the current study (Enevoldsen et al., 2007; Lange, 2004; Rognstad & Katz, 1966; Yoshioka et al., 2001).

In the genome‐wide analysis between exercise‐induced changes in gene expression and the exercise‐induced increase in serum GH, the strongest associations were found for *ARHGAP24*, *TAF4B*, *ARID5B*, *MIR604* and *MIR938* mRNA, all directly related to the increase in serum GH (Figure 6). Interestingly, three of these genes—*ARID5B*, *MIR604* and *MIR938*—have been found to be upregulated in AT after a bolus injection of GH (Høyer et al., 2020) supporting a causal relationship between the exercise‐induced increase in serum GH and changes in their expression. The functions of these genes are not well understood, but some direct relationship to AT metabolism or an indirect relationship through TGFB, which in turn interacts with inflammation and lipolysis, has been suggested (Zhang et al., 2024). ARHGAP24 may reflect decreased TGFB signalling (Motizuki et al., 2021), TAF4B interacts with TGFB in osteogenic cells (Iwata et al., 2010), and MIR938 regulates TGFB in pituitary adenomas (Butz et al., 2011). MIR604 regulates FASN in mouse heart (Weldy et al., 2020). ARID5B is known from mouse experiments to be involved in AT metabolism, with increased expression described as depressing lipolysis and thermogenesis (Whitson Jr. et al., 2021). This is opposite the expected effect, given the direct association between exercise‐induced increases in GH and *ARID5B* mRNA. However, AT metabolism is complex, and counter‐regulatory mechanisms are necessary to restrict excessive lipolysis (Yang & Mottillo, 2020). This is comparable to the role of CISH in controlling GH‐induced lipolysis (Møller & Jørgensen, 2009).

### 4.4 Adipose tissue stress: Early versus late post‐exercise period

We previously reported that genes related to mitochondrial function and fat metabolism in AT were downregulated, while some inflammation markers were upregulated 15 min after acute SIT (Esbjörnsson, Rundqvist, et al., 2024). In contrast, 2 h post‐SIT in the present study, mitochondrial‐related gene expression was unchanged, while upstream regulator analysis predicted an anti‐inflammatory response, with activation of TGFBR2 and inactivation of NFKB1 (Lee, 2018). This apparent shift from an early stress response to a more functional pattern may reflect several mechanisms. At SIT onset, sympatho‐adrenergic activity and AT lipolysis rapidly increase (Esbjörnsson‐Liljedahl et al., 2002; Esbjörnsson, Rundqvist, et al., 2024), while AT blood flow decreases (Figure 3), potentially promoting FFA entrapment, as supported by the transient fall and subsequent rebound in plasma FFA (Figure 2). Reduced blood flow together with increased lipolysis may create a transient hypoxic, pro‐stress environment (Lipke et al., 2022), consistent with early post‐exercise reductions in AT mitochondrial gene expression, hypoxanthine accumulation in AT and increased IL‐6 in an AT vein (Esbjörnsson, Norman, et al., 2024; Esbjörnsson, Rundqvist, et al., 2024). Yet, short‐term AT stress may subsequently promote an anti‐inflammatory response (Gleeson et al., 2011). Potential mediators include IL‐6, succinate/SUCNR1 and glutamine (Huang et al., 2024; Keiran et al., 2019; Liebing & Staubert, 2026; Luan et al., 2023; Macpherson et al., 2015; Petrus et al., 2020). In the present study, SUCNR1 mRNA increased after SIT (unadjusted *p* = 0.003; Figure S1), but not CON, and was positively associated with plasma FFA 2 h post‐exercise. Previously, we observed an increased glutamine‐to‐glutamate ratio in AT after acute SIT (Esbjörnsson et al., 2006). Collectively, these findings are hypothesis‐generating and suggest that SIT may trigger a transition from an early stress response to a later anti‐inflammatory state in AT. Mechanistic studies are needed to confirm these pathways at the protein and functional signalling levels in human AT.

An anti‐inflammatory AT response was also predicted from upstream regulators in CON (Table S2). This suggests that, for example, diurnal rhythms also drive pathways common to those related to exercise (Loboda et al., 2009; Pendergrast et al., 2023), and a complex interaction between diurnal rhythms and exercise‐specific effects has been demonstrated in mouse AT (Pendergrast et al., 2023). Nevertheless, the increased IL‐6 release from AT and HPX accumulation in AT were related to plasma lactate accumulation and ATP depletion in skeletal muscle as an early response to SIT (Esbjörnsson, Norman, et al., 2024; Esbjörnsson, Rundqvist, et al., 2024). This implies exercise‐specific effects in AT, in addition to other drivers of AT metabolism. An exercise‐induced crosstalk between immune cells and adipocytes has recently been demonstrated in humans (Dollet et al., 2024).

### 4.5 Earlier studies of acute exercise and AT gene expression

To our knowledge, only one previous publication has examined global AT gene expression after SIT (Esbjörnsson, Rundqvist, et al., 2024). However, there are some studies on the regulation of abdominal subcutaneous AT gene expression at exercise intensities ranging from 30% to 90% of VO2max, reporting between 10 and 4000 DEGs (Ahn et al., 2025; Dreher et al., 2023; Fabre et al., 2018; Ludzki et al., 2021), with no obvious relationship between exercise intensity and the number of DEGs. The inflammatory response, as assessed by DEGs, gene set enrichment, or upstream analysis, is inconsistent across these studies. Some reported an inflammatory response (Ahn et al., 2025; Fabre et al., 2018; Ludzki et al., 2021), while one of them found an anti‐inflammatory response (Dreher et al., 2023). None of the previous studies reported upregulation of genes related to lipolysis or thermogenesis.

Differences in study populations, such as obese/non‐obese, or sedentary/trained individuals, sex, experimental protocols including exercise modality and intensity, fasting or non‐fasting state, timing of post‐exercise biopsies, number of biopsies and time of day, could explain the considerable variation in gene expression outcomes in earlier exercise studies on AT (Ahn et al., 2025; Dreher et al., 2023; Fabre et al., 2018; Guerrier et al., 2023; Loboda et al., 2009; Ludzki et al., 2021). The number of DEGs in the current study of non‐obese subjects was at the lower end of the range reported in earlier studies of acute exercise on AT. After correcting for background drivers of gene expression, such as time of day, by comparing SIT and CON, an even lower number of DEGs were identified. The lack of non‐exercise controls may contribute to the heterogeneous responses observed when comparing different studies.

### 4.6 Sex differences in gene expression at rest and in response to SIT

In the present study, an equal number of males and females were included, although there were no specific sex‐related research questions. Due to the low number of individuals in CON (3 males and 3 females) the statistical power was likely too low to identify sex differences in DEGs, especially given possible interference from diurnal variations in AT gene expression. However, sex is an important determinant of AT metabolism at rest, after a meal, or during exercise (Clegg & Mauvais‐Jarvis, 2018; Costa et al., 2025; Massier et al., 2025).

Regarding SIT, several sex‐related systemic changes could potentially affect the AT transcriptome. For example, females reached the level of GH peak faster than males during SIT in the present and previous studies (Esbjörnsson et al., 2009; Rundqvist et al., 2019). Moreover, in females, elevated serum GH levels were observed already pre‐exercise in both SIT and CON, possibly being an anticipatory response to exercise (Eliakim, Cooper, & Nemet, 2014; Eliakim, Nemet, et al., 2014; Engström et al., 1998; Esbjörnsson et al., 2009; Rundqvist et al., 2019; Wideman et al., 1999, 2002). In line with this sex‐related difference in serum GH profile, the GH‐responsive gene *CISH* (Vestergaard et al., 2014) also showed several‐fold higher expression pre‐exercise in females than in males (Figure 7). This suggests that the sex difference in serum GH is also reflected at the gene transcriptional level.

Subjects were randomly assigned to SIT or CON just prior to the onset of exercise, which might explain an anticipatory response in both SIT and CON. The response to exercise also differed between males and females for *CISH* mRNA. In the SIT group, males showed an increase and females a decrease in *CISH* mRNA (time × sex; unadjusted *p* = 0.003). A similar pattern was observed for the GH‐associated genes *MIR604* and *MIR938* (time × sex; unadjusted *p* = 0.030 and *p* = 0.038, respectively).

### 4.7 Acute response to SIT—Skeletal muscle versus adipose tissue gene expression

Exercise‐induced changes in gene expression in skeletal muscle were not analysed in the present study but were examined in a previous study using a similar SIT protocol as in the current one (Rundqvist et al., 2019). We found a clearly divergent response of skeletal muscle to acute SIT compared to that in AT, consistent with an earlier report (Dreher et al., 2023). The number of DEGs was more than 10‐fold higher in skeletal muscle than in AT. Examples of activated upstream regulators in the skeletal muscle include factors related to inflammation, such as transcription factors, cytokines, growth factors and insulin. In AT, such factors were predicted to be inactivated (e.g. SMAD3, NFKB1, TNF, EGF and insulin). One factor in common to both skeletal muscle and AT was the predicted activation of GH. A discordant pattern between adipose tissue and skeletal muscle gene expression was also observed after strength exercise combined with endurance training (Svensson et al., 2024). The divergent response of skeletal muscle and AT was not unexpected, given the profound differences in the function of these two tissues. However, some associations in the acute response of energy‐related metabolites between these tissues have been described previously (Esbjörnsson, Rundqvist, et al., 2024). One possible common factor is the systemic hormonal response. One example is the response of *CISH* mRNA to acute SIT. Females had higher pre‐exercise levels than males in both tissues, like the pronounced sex difference observed in serum GH in a pre‐exercise situation (Esbjörnsson et al., 2009). Regarding the response of *CISH* mRNA to SIT, males had a larger response than females in both skeletal muscle (Rundqvist et al., 2019) and AT (Figure 7).

### 4.8 Strength and limitations

A strength of the present study is the inclusion of a control group performing unloaded exercise (CON). This is important for distinguishing the effects of SIT from other potential drivers of gene expression in adipose tissue (AT), such as diurnal rhythms and fasting. Another strength is the balanced representation of males and females, although this may also have increased interindividual variability in gene expression. Furthermore, we studied a young, healthy, non‐obese population, like the population in which SIT‐induced post‐exercise energy expenditure was previously demonstrated (Hazell et al., 2012). Finally, a notable strength is the use of a well‐characterised experimental protocol for investigating the response to SIT, which has also been applied in our previous studies (Esbjörnsson, Rundqvist, et al., 2024).

The study also has several limitations. First, the study was likely underpowered, particularly because of the unequal group sizes (6 participants in CON and 12 in SIT), to detect between‐group differences in the associations between exercise‐induced changes in gene expression and GH. This was supported by a post hoc sensitivity power analysis. The smaller sample size in CON was chosen to minimise the number of participants undergoing invasive procedures, as we did not expect substantial changes in gene expression in this condition. Participants were informed of their allocation to CON or SIT immediately before the start of cycling. This may have elicited an anticipatory GH response in CON, potentially influencing the GH outcomes. Finally, diurnal variation in gene expression may have contributed to the changes observed in CON. To the best of our knowledge, this is the first study to investigate the acute transcriptomic response of adipose tissue to exercise using a unload‐exercise control group. Second, only two biopsies were performed, before and 2 h after exercise, which limits the temporal resolution of the gene expression response. However, limiting the number of biopsies can also be considered a strength, as repeated biopsies may induce an inflammatory response that could affect the measured outcomes (Fabre et al., 2018). The 2‐h post‐exercise time point was selected based on previous studies demonstrating adipose tissue responses to exercise and GH within 1–3 h (Enevoldsen et al., 2007; Hjelholt et al., 2019; Høyer et al., 2020). Nevertheless, a stronger response at 3 h than at 2 h post‐exercise cannot be fully excluded. Third, the biopsies were limited to abdominal subcutaneous AT. However, as this depot is often the single largest AT depot in the body, it is likely to make a substantial contribution to whole‐body lipolysis in humans (Frayn & Humphreys, 2012; Massier et al., 2025). Fourth, although adipocytes constitute most of the volume of adipose tissue, AT also contains numerous other cell types, including cells associated with the vasculature and immune system. Consequently, changes occurring specifically in adipocytes may to some extent be masked by signals originating from other cell types. Finally, AT analyses were performed only at the mRNA level. Future studies should therefore confirm the present findings at the protein and functional levels to provide a more comprehensive understanding of how GH stimulates lipolysis in AT following acute SIT. In addition, quantitative real‐time PCR validation of the top‐ranked genes identified in the genome‐wide analysis could have strengthened the findings and provided further confirmation of the observed gene expression changes and associations with the GH‐response to acute SIT.

## 5 CONCLUSION

Following acute sprint interval exercise (SIT), we found no major enrichment of differentially expressed genes related to lipolysis or thermogenesis in abdominal subcutaneous adipose tissue (AT). However, an interaction was observed between the exercise‐induced change in *GHR* mRNA between SIT and controls with unloaded exercise (CON), supporting the demonstrated associations between exercise‐induced changes in GH‐responsive genes in AT and circulatory GH. This partly confirmed our secondary hypothesis. Nevertheless, the observation of changes in AT gene expression also in CON raised important methodological concerns. Clock genes in AT are known to regulate genes involved in energy metabolism (Loboda et al., 2009). Sex was also found to influence both the circulatory GH response to exercise and, for example, the well‐known GH‐responsive gene *CISH*. This reinforces, from a molecular perspective, the importance of controlling for diurnal variation and sex in exercise studies on regulation of AT‐lipolysis.

## AUTHOR CONTRIBUTIONS

**Mona Esbjörnsson:** Conceptualization; data curation; formal analysis; funding acquisition; investigation; methodology; project administration; resources; software; supervision; validation; visualization. **Håkan C. Rundqvist:** Formal analysis; investigation; methodology; software; visualization. **Barbara Norman:** Conceptualization; data curation; investigation; methodology; visualization. **Ted Österlund:** Investigation; methodology; software; visualization. **Jens Bülow:** Conceptualization; investigation; methodology; resources. **Eva Jansson:** Conceptualization; data curation; formal analysis; funding acquisition; investigation; methodology; project administration; resources; software; supervision; validation; visualization.

## FUNDING INFORMATION

This study was supported by grants from the Swedish Research Council for Sport Science (P2013‐0101 and FO2014‐0009), the Swedish Society of Medicine, Center of Gender Related Medicine, Stiftelsen Sigurd & Elsa Goljes Minne (LA2022‐0092) and O.E. och Edla Johanssons Vetenskapliga Stiftelse.

## CONFLICT OF INTEREST STATEMENT

The authors declare no conflict of interest.

## ETHICS STATEMENT

Subjects were fully informed about the procedures and potential risks of the experiment before giving written informed consent to participate in the study, which was approved by the Ethics Committee at Karolinska Institutet (Dnr 2015/574‐31, 2015/1054‐32, 2015/1646‐32, 2015/2340‐32).

## Supporting information


**Figure S1.** (a) Plasma free fatty acids (FFA) 2 h post‐exercise related to serum GH peak, (b) exercise‐induced changes of *SUCNR1* gene expression (2 h post exercise ‐ pre exercise) related to increase in serum GH (GH peak ‐ pre) in 18 subjects with SIT (all‐out bouts) and CON (unloaded exercise) and (c) *SUCNR1* gene expression pre‐exercise and 2 h post‐exercise divided by load (SIT, 6 females and 6 males) and CON, 3 females and 3 males) and sex. *SUCNR1* data were log2‐transformed and are mean ± SE.

**Figure S2.** Exercise‐induced fold changes (FC) in SIT (12 subjects) versus CON (6 subjects). The 77 included genes were obtained at a cut‐off of adjusted *p* < 0.3 for the SIT group in the *limma* analysis.

**Table S1.** Differentially expressed genes expressed as fold change (FC; post/pre; FC <1 is expressed as neg FC) in subcutaneous adipose tissue in SIT (6 females and 6 males). Data were analysed by *limma*.

**Table S2.** Differentially expressed genes expressed as fold change (FC; post/pre; FC <1 is expressed as neg FC) in subcutaneous adipose tissue in CON (3 females and 3 males). Data were analysed by *limma*.

**Table S3.** Differentially expressed genes expressed as fold change (FC; post/pre; FC <1 is expressed as neg FC and adjusted *p*‐values) in subcutaneous adipose tissue in SIT (6 females and 6 males) and CON (3 females and 3 males) and interaction between SIT and CON with P and adjusted P. Data were analysed by *limma*.

**Table S4.** Differentially expressed genes expressed as fold change (FC; post/pre; FC <1 is expressed as neg FC) in subcutaneous adipose tissue in SIT and CON combined (9 females and 9 males). Data were analysed by *limma*.

**Table S5.** Upstream regulators as identified by the IPA‐analysis from 77 differentially expressed genes (adjusted *p* < 0.3) in subcutaneous adipose tissue in SIT (6 females and 6 males).

**Table S6.** Upstream regulators as identified by the IPA‐analysis from 136 genes (fold change cut off ±1.6) in subcutaneous adipose tissue in CON (3 females and 3 males).

**Table S7.** Upstream regulators as identified by the IPA‐analysis from 110 differentially expressed genes (adjusted *p* < 0.1) in subcutaneous adipose tissue in SIT and CON combined (9 females and 9 males).

**Table S8.** Correlations between serum GH peak (adjusted for pre‐values) and post‐exercise gene expression (array data, adjusted for pre‐values) in SIT and CON groups (9 females and 9 males).

## Data Availability

The gene expression datasets generated and analysed during the current study have been deposited in the Gene Expression Omnibus (GEO) database at the National Center for Biotechnology Information (NCBI) and are accessible through GEO Series accession number GSE335793. Other underlying datasets supporting the findings of this study are not publicly deposited but are available from the corresponding author upon reasonable request.
