# Supplementary material for: Acute sprint exercise transcriptome in adipose tissue of healthy adults: Associations with growth hormone
Source: Physiol Rep. 2026 Nov 6;14(21):e71160. doi: 10.14814/phy2.71160 (PMC13740967; doi:10.14814/phy2.71160)
Supplement: Supplementary file 1 [file PHY2-14-e71160-s005.pdf]

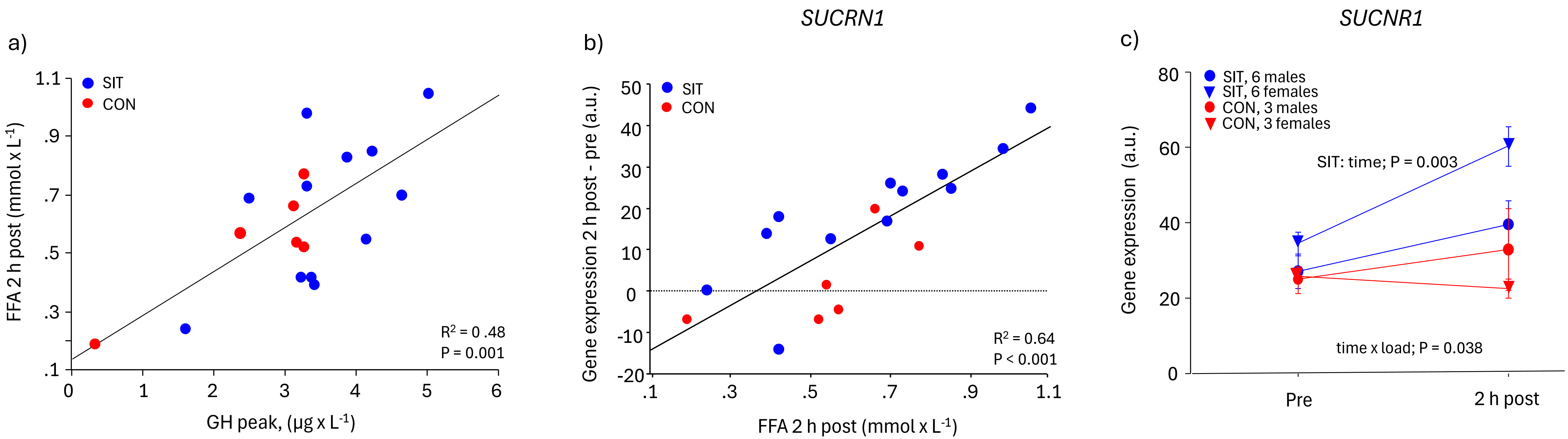

a)
SIT
CON
1.1
.9
.7
.5
.3
.1
0
1
2
3
4
5
6
FFA 2 h post (mmol x L-1)
GH peak, (µg x L-1)
R2 = 0 .48
P = 0.001
SUCRN1
b)
SIT
CON
50
40
30
20
10
0
-10
-20
.1
.3
.5
.7
.9
1.1
Gene expression 2 h post - pre (a.u.)
FFA 2 h post (mmol x L-1)
R2 = 0.64
P < 0.001
SUCNR1
c)
SIT, 6 males
SIT, 6 females
CON, 3 males
CON, 3 females
80
60
40
20
0
SIT: time; P = 0.003
time x load; P = 0.038
Gene expression (a.u.)
Pre
2 h post
