# Supplementary material for: Acute sprint exercise transcriptome in adipose tissue of healthy adults: Associations with growth hormone
Source: Physiol Rep. 2026 Nov 6;14(21):e71160. doi: 10.14814/phy2.71160 (PMC13740967; doi:10.14814/phy2.71160)
Supplement: Supplementary file 2 [file PHY2-14-e71160-s007.pdf]

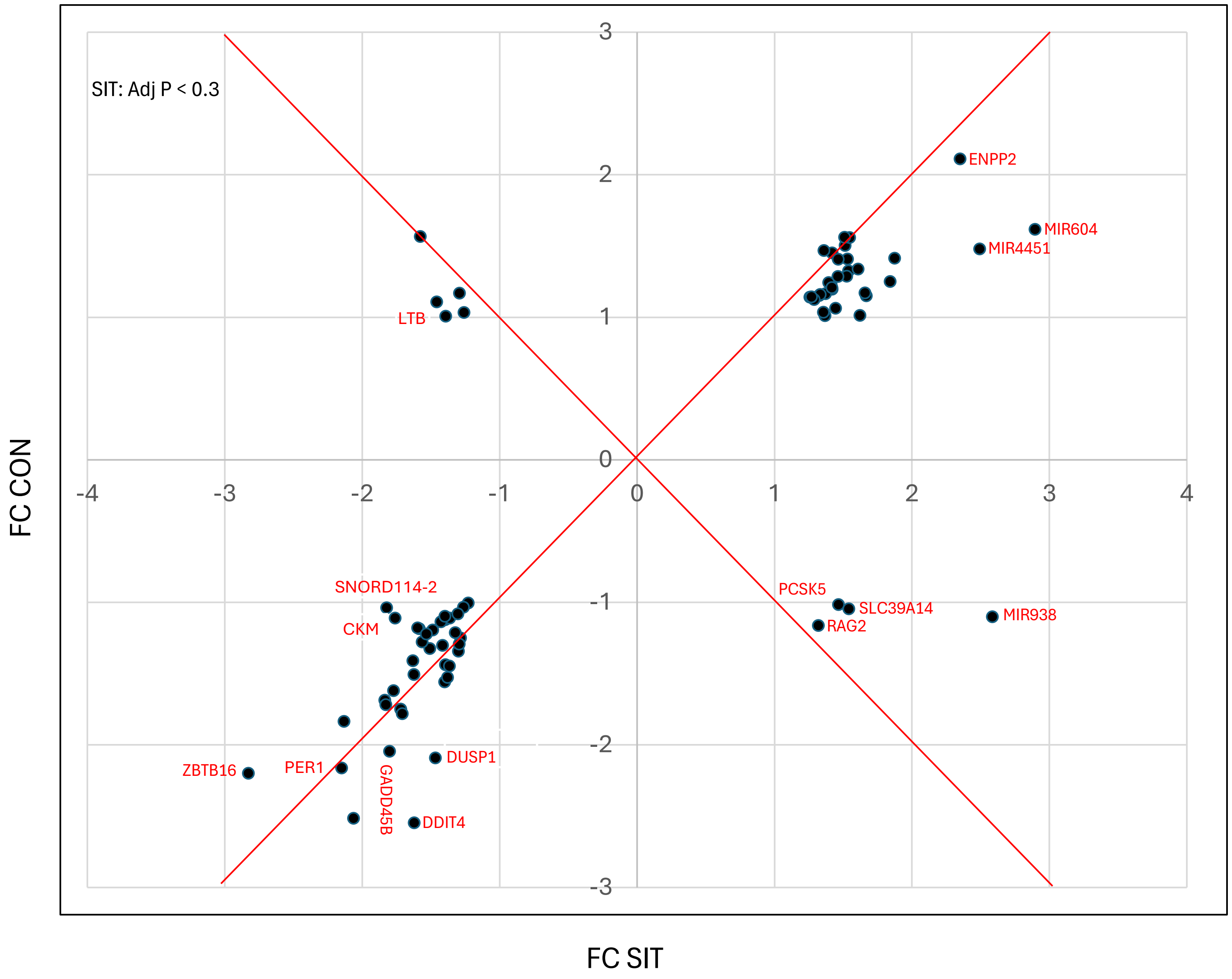
SIT: Adj P < 0.3
ENPP2
MIR604
MIR4451
LTB
SNORD114-2
CKM
PCSK5
SLC39A14
RAG2
MIR938
ZBTB16
PER1
GADD45B
DUSP1
DDIT4
FC CON
FC SIT
